# Analysis of gut microbiota characteristics in osteoarthritis patients

**DOI:** 10.3389/fcimb.2026.1915556

**Published:** 2026-08-06

**Authors:** Peng Zou, Xuejian Liu, Yichong Wang, Zhichao Xue, Jianxin Yang, Fangfang Meng, Nan Dong

**Affiliations:** The Department of Orthopedics, The Seventh People’s Hospital of Zhengzhou, Institute of Biological Therapy, Henan Academy of Innovations in Medical Science, Zhengzhou, China

**Keywords:** gut bacterial microbiota, gut fungal microbiota, gut-bone axis, inflammatory factors, osteoarthritis

## Abstract

**Objective:**

Osteoarthritis (OA) is a chronic and debilitating disease that represents a leading cause of disability among middle-aged and elderly individuals. With the aging population, the incidence of OA and its associated socioeconomic burden is increasing; however, the underlying pathogenesis remains unclear. Based on the “gut-bone axis” theory, this study systematically characterized the gut microbiota of patients with OA to identify potential biological targets for clinical diagnosis and treatment.

**Methods:**

Fecal samples from 32 OA patients and 26 healthy controls were analyzed for gut bacterial and fungal microbiota using 16S rRNA and ITS high-throughput sequencing. ELISA measured serum levels of inflammatory biomarkers. Spearman’s rank correlation coefficient was used to assess relationships between gut microbiota and inflammatory markers. Random forest and receiver operating characteristic (ROC) analyses were applied to identify characteristic microbial taxa associated with OA.

**Results:**

Serum levels of the inflammatory factors LPS, IL-1β, IL-6, and IL-10 were significantly elevated in OA patients. Both alpha diversity and overall composition of the gut bacterial and fungal microbiota were altered in OA patients. At the phylum level, the abundance of *Proteobacteria* increased significantly, whereas that of *Firmicutes* and *Bacteroidota* decreased markedly. At the genus level, the abundances of *Escherichia-Shigella* and the *Ruminococcus gnavus* group were significantly increased, while those of *Bacteroides*, the *Eubacterium halli* group, *Subdoligranulum*, and *Faecalibacterium* were significantly decreased. Regarding gut fungi, the phyla *Ascomycota* and *Basidiomycota* were significantly enriched in OA patients. At the genus level, *Saccharomyces* and *Aureobasidium* were significantly more abundant, whereas *Aspergillus*, *Exophiala*, and *Kurtzmaniella* were significantly reduced. Cross-kingdom network analysis revealed weakened interactions between gut bacteria and fungi in OA patients, disrupting the overall ecological balance of the gut microbiome. Random forest and ROC analyses indicated that the combination of the *Eubacterium coprostanoligenes* group, *Subdoligranulum*, the *Ruminococcus gauvreauii* group, *Malassezia*, and *Aspergillus* exhibited strong diagnostic potential for OA. These microbial changes were closely correlated with inflammatory markers.

**Conclusion:**

OA patients present with both elevated serum inflammatory factors and gut microbial dysbiosis, and significant associations exist between specific microbiota and inflammatory markers. While these findings suggest that certain gut microbes could serve as potential diagnostic biomarkers for OA, further validation is necessary to confirm their clinical applicability.

## 1 Introduction

Osteoarthritis (OA) is the most common joint disorder in clinical practice, affecting over 500 million people worldwide and representing a leading cause of disability among middle-aged and elderly individuals. As the population ages, the socioeconomic burden of OA on both China and the global community will continue to increase (Li et al., 2025). For a long time, OA has been regarded as a “wear-and-tear” disease caused by excessive mechanical loading and joint instability, with progressive cartilage damage as its primary clinical and pathological feature. However, this mechanical wear hypothesis fails to explain why many patients with severe radiographic cartilage degeneration experience no obvious pain, nor does it account for the limited efficacy of treatments targeting purely mechanical factors.

Recent studies have identified chronic, persistent inflammation as a central driver of OA onset and progression. Elevated levels of pro-inflammatory cytokines (e.g., IL-1β, TNF-α, and IL-6), chemokines, and matrix metalloproteinases have been reported in the synovium, joint fluid, and even chondrocytes of OA patients. These inflammatory mediators not only directly induce chondrocyte apoptosis and extracellular matrix degradation but also amplify inflammatory effects through positive feedback loops, thereby promoting OA progression (Kapoor et al., 2011; Wojdasiewicz et al., 2014; Negishi et al., 2024). Nevertheless, key questions remain: where does local joint inflammation originate, and what factors continuously drive the development of OA-related inflammation? Answering these questions poses a critical challenge for researchers aiming to develop drugs capable of preventing or delaying OA progression.

The “gut-bone axis” is an important concept developed in recent years, proposing bidirectional communication between the gut microbiota and the skeletal system via immune, metabolic, and neuroendocrine pathways (Li et al., 2024; You et al., 2025; Zhang et al., 2026). Dysbiosis of the gut microbiota can impair intestinal barrier function, allowing bacteria and their metabolites to translocate into the bloodstream, triggering systemic low-grade inflammation that subsequently affects the joint microenvironment and promotes OA development (Ramires et al., 2022; Xi et al., 2025). Several recent studies have demonstrated a close association between gut microbiota and OA pathogenesis. Guan et al. reported that antibiotic-induced gut dysbiosis alleviated OA progression (Guan et al., 2023). Ulici et al. showed that germ-free mice exhibited reduced severity of OA induced by medial meniscus instability compared with conventional mice, suggesting that gut microbiota-related factors promote OA development following joint injury (Ulici et al., 2018). Clinical data further supports this link. Huang et al. found that serum and synovial fluid LPS levels, originating from the gut microbiota, correlate with knee OA severity and inflammation (Huang et al., 2026). Other researchers have reported that the abundance of *Streptococcus* species is associated with knee pain in OA patients (Boer et al., 2019). Another study revealed a significant positive correlation between the degree of gut dysbiosis and knee pain scores, joint space narrowing, and MRI findings of synovitis in OA patients (de Sire et al., 2025). Collectively, these findings highlight the critical role of gut microbiota in OA pathogenesis. However, which specific microbial taxa play key roles in promoting OA onset and progression remains unclear, and no consensus has been reached within the scientific community.

To elucidate the characteristics of the gut microbiota in OA patients, this study employed 16S rRNA and ITS high-throughput sequencing to analyze the gut microbiome of hospitalized OA patients and compared it with that of healthy controls, aiming to identify core microbial communities associated with OA. The results provide theoretical foundations and data support for further research into the mechanisms underlying OA development and potential therapeutic interventions.

## 2 Materials and methods

### 2.1 Study protocol and ethical statement

A total of 32 patients with active knee osteoarthritis (OA) and 26 healthy controls (HC) were enrolled in this study. All OA patients were recruited from those admitted to the Department of Orthopedics at Zhengzhou Seventh People’s Hospital between October 2024 and December 2025. Healthy controls were recruited from individuals undergoing routine physical examinations at the same hospital during the same period. All patients met the diagnostic criteria of the Chinese Guidelines for the Diagnosis and Treatment of Osteoarthritis (2021 Edition). Exclusion criteria for OA patients were as follows: (i) use of antibiotics, prebiotics, non-steroidal anti-inflammatory drugs (NSAIDs), or microecological preparations within one month prior to enrollment; (ii) use of statins within the same period; (iii) presence of severe hepatic or renal dysfunction, diabetes mellitus, or malignant tumors; (iv) diagnosis of other inflammatory joint diseases (e.g., rheumatoid arthritis, gout, or psoriatic arthritis), inflammatory bowel disease, irritable bowel syndrome, or a history of gastrointestinal surgery. Inclusion criteria for healthy controls included: (i) age- and sex-matched to the OA cohort; (ii) absence of the aforementioned inflammatory joint diseases, inflammatory bowel disease, irritable bowel syndrome, or gastrointestinal surgery history; (iii) absence of severe hepatic/renal dysfunction, diabetes, or malignancies; (iv) no use of antibiotics, prebiotics, NSAIDs, statins, or microecological preparations within one month before enrollment. Basic demographic and clinical data were collected at the time of the first enrollment assessment. Human fecal and blood sample collection and all experimental procedures were approved by the Ethics Review Committee of Zhengzhou Seventh People’s Hospital (Approval No: ky-027) in accordance with the guidelines provided by the Chinese Health Science Administration. Written informed consent was obtained from all participants in compliance with the Declaration of Helsinki.

### 2.2 Sample collection and fecal DNA extraction

Blood samples were collected aseptically from all participants and centrifuged at 3, 000 rpm for 10 minutes at 4 °C within 30 minutes of collection to separate serum. The resulting serum aliquots were immediately transferred to sterile cryovials and stored at −80 °C until further analysis. Fresh fecal samples were aseptically collected on the first day of admission and stored at −80 °C for subsequent DNA extraction. Total genomic DNA was extracted from fecal samples using a DNA extraction kit (TiGen, China) following the manufacturer’s instructions. DNA quality and concentration were assessed using a NanoDrop^®^ ND-2000 spectrophotometer (Thermo Scientific Inc., USA) and 1.0% agarose gel electrophoresis. All DNA samples were then sent to a commercial sequencing company for 16S rRNA and ITS sequencing.

### 2.3 Gut microbiota analysis

To characterize the bacterial gut microbiota, PCR amplification of the V3–V4 hypervariable region of the 16S rRNA gene was performed using universal primers 338F (5’-ACTCCTACGGGAGGCAGCAG-3’) and 806R (5’-GGACTACNNGGGTATCTAAT-3’). To characterize the fungal gut microbiota, PCR amplification of the internal transcribed spacer 2 (ITS2) region was performed using universal primers ITS3F (5’-GCATCGATGAAGAACGCAGC-3’) and ITS4R (5’-TCCTCCGCTTATTGATATGC-3’). The PCR amplicons were purified using an AxyPrep DNA Gel Extraction Kit and quantified using a Quantus™ fluorometer. Sequencing was performed on the MiSeq PE300 platform (Illumina, San Diego, CA, USA) by Majorbio Bio-Pharm Technology Co., Ltd. (Shanghai, China). Sequencing data were analyzed using the Majorbio Cloud online platform (https://cloud.majorbio.com).

### 2.4 Measurement of inflammatory biomarkers in serum

Serum levels of inflammatory biomarkers, including LPS, IL-1β, IL-6, and IL-10, were measured using enzyme-linked immunosorbent assay (ELISA) according to the manufacturer’s instructions. All commercial ELISA kits were purchased from Shanghai Enzyme-linked Biotechnology Co., Ltd. (Shanghai, China).

### 2.5 Bioinformatics analysis

Bioinformatics analysis of the gut microbiota was performed using the Majorbio Cloud online platform (https://cloud.majorbio.com). Alpha diversity of the gut microbiota was assessed using the Chao1 and Simpson indices, while beta diversity was calculated based on principal coordinate analysis (PCoA) using unweighted UniFrac distances. Differences in gut microbiota composition between groups were analyzed using PERMANOVA and the Wilcoxon rank-sum test. Differences in gut microbiota at the genus level between groups were identified using LEfSe analysis. Spearman’s rank correlation coefficient was used for correlation analyses. Linear regression analysis was performed to evaluate associations between major bacterial genera, fungal genera, and cytokines. Cross-domain network diagrams illustrating interactions between bacterial and fungal genera were generated using the igraph package (version 1.2.6) in R software.

### 2.6 Statistical analysis

All statistical analyses were performed using GraphPad Prism version 10.4.1. For data following a normal distribution, independent-sample t-tests were used to compare parameters between groups. For data not following a normal distribution, Mann-Whitney Rank Sum Test were applied. A *p*-value of less than 0.05 was set as the threshold for statistical significance. Linear regression analysis and ROC curves were generated using GraphPad Prism (version 10.4.1). The area under the curve (AUC) and its 95% confidence interval (CI) were calculated to identify biomarkers for distinguishing OA patients from healthy controls.

## 3 Results

### 3.1 Demographic characteristics of the study participants

All participants in this study were of Han Chinese ethnicity from Henan Province and shared similar dietary habits. A total of 58 participants were enrolled, including 26 healthy controls and 32 OA patients. The OA group comprised 14 males and 18 females, with a mean age of 60.59 years. The healthy control group comprised 12 males and 14 females, with a mean age of 58.65 years. No significant differences in demographic characteristics, including age, sex ratio, or body mass index (BMI), were observed between the two groups (**Table 1**).

**Table 1:** Clinical characteristics of subjects in each group.

Characteristics	HC group (n=26)	OA group (n=32)	P-value
Sex			
Male	12(46.15%)	14(43.75%)	>0.9999
Female	14(53.85%)	18(56.25%)	>0.9999
Age (years)	58.65 ± 4.71	60.59 ± 4.92	1.337
Weight (kg)	72.93 ± 9.76	77.22 ± 7.36	0.0618
BMI	24.03 ± 3.39	25.79 ± 3.30	0.0511

Values are mean ± SD for continuous variables. Significance was assessed using an unpaired t-test. OA, osteoarthritis. HC, health control group.

### 3.2 Serum levels of inflammatory factors in OA patients

**Figure 1:**
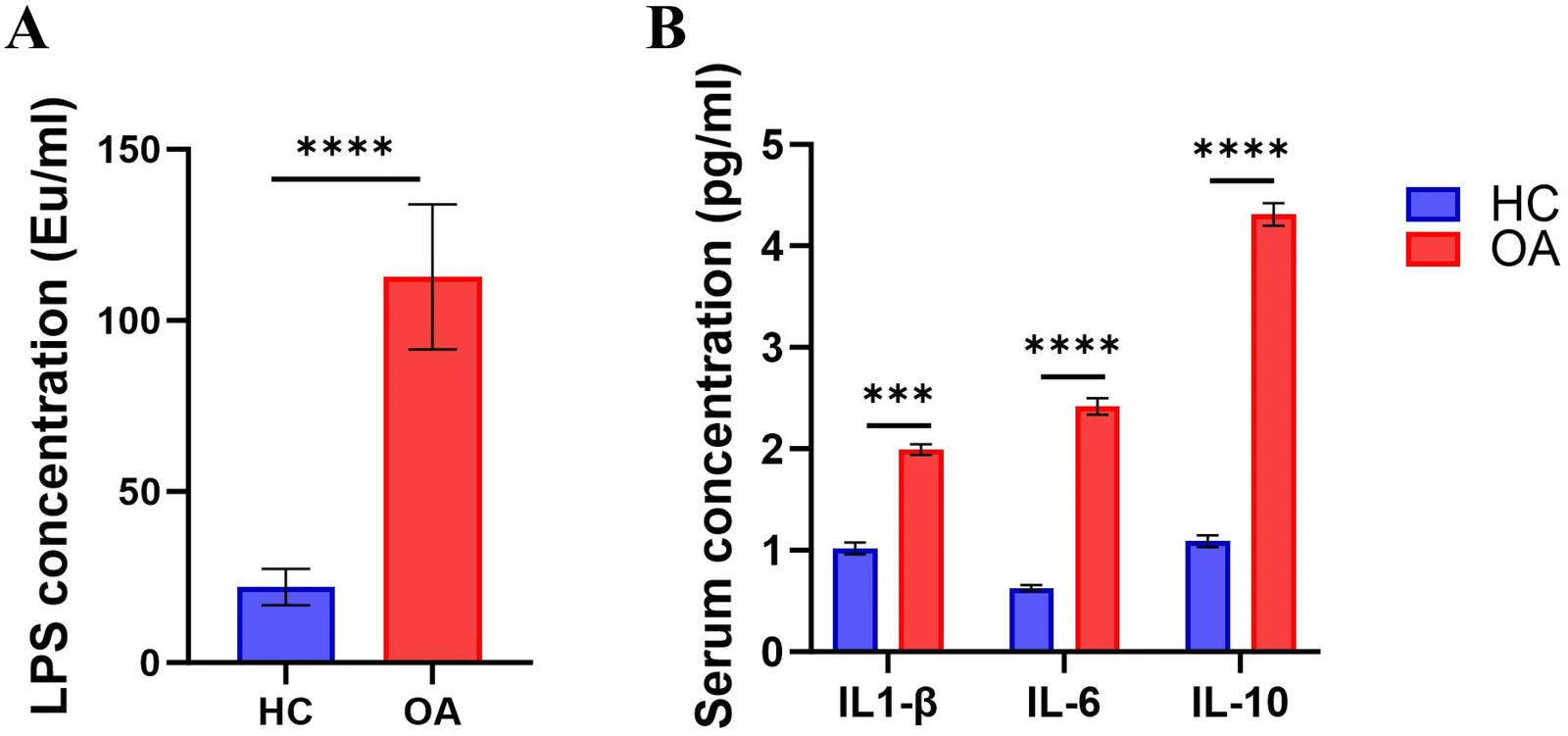
Serum inflammatory factor levels of OA patients and the HC group. **(A)** Comparison of serum LPS levels between OA patients and the HC group. **(B)** Serum IL-1β, IL-6, and IL-10 levels between OA patients and the HC group. Data are represented as means ± SD. Significance was assessed using an unpaired t-test. OA, osteoarthritis (n=32). HC, health control group (n=26). ***p < 0.001; ****p < 0.0001.

We measured serum levels of LPS and inflammatory cytokines, including IL-1β, IL-6, and IL-10, in OA patients and healthy controls. The results showed that the mean serum LPS concentration in OA patients was 112.70 EU/mL, compared with 22.09 EU/mL in the HC group, with a significant difference between the two groups (*p* < 0.0001, **Figure 1A**). Furthermore, serum levels of IL-1β, IL-6, and IL-10 were significantly elevated in OA patients compared with healthy controls (**Figure 1B**). Collectively, these results indicate that OA patients exhibit high levels of inflammatory markers.

### 3.3 Analysis of gut microbiota in OA patients

**Figure 2:**
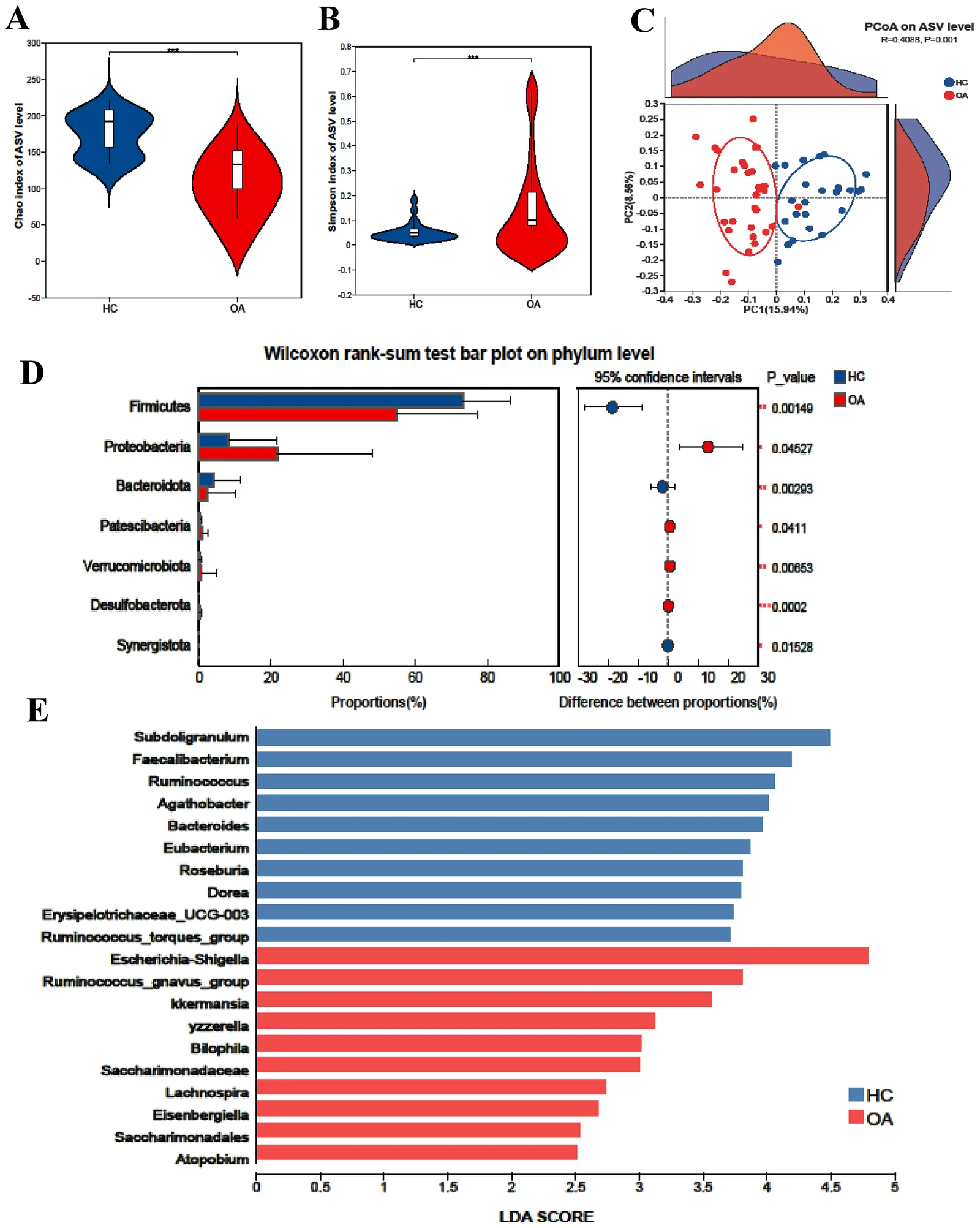
Comparison of gut microbiota between OA patients and healthy controls. **(A)** Chao index. **(B)** Simpson index. **(C)** PCoA analysis based on unweighted UniFrac distance at the ASV level. ANOSIM was used to analyze the differences in the bacterial community between the OA group and the HC group. **(D)** Comparisons of the relative abundances of gut microbiota at the phylum level between the OA and HC groups with the Wilcoxon rank-sum test. **(E)** The LEfSe analysis of gut microbiota at the genus level between the OA and HC groups (LDA>2.5, *P* < 0.05). OA, osteoarthritis (n=32). HC, health control group (n=26). LEfSe, linear discriminant analysis effect size; LDA, linear discriminant analysis; Statistical differences were calculated using the Wilcoxon rank-sum test for two groups. *p < 0.05, **p < 0.01, ***p < 0.001.

Alpha diversity of the gut bacterial microbiota was assessed using the Chao1 and Simpson indices. The Chao index reflects community richness, while the Simpson index reflects community diversity. Compared with healthy controls, the OA group exhibited a significantly lower Chao index (**Figure 2A**) and a significantly higher Simpson index (**Figure 2B**), indicating that gut bacterial alpha diversity was significantly reduced in OA patients. Beta diversity of the gut bacterial microbiota was evaluated using PCoA based on the abundance-weighted Jaccard distance at the ASV level. The results showed that samples from the OA and HC groups clustered in distinct regions of the coordinate space, and ANOSIM revealed a significant difference between the two groups (*p* = 0.001, **Figure 2C**), suggesting that OA alters the structure of the gut bacterial community. Differences in gut bacterial composition between the two groups were assessed at various taxonomic levels. At the phylum level, OA patients showed a significantly increased abundance of *Proteobacteria* and significantly decreased abundances of *Firmicutes* and *Bacteroidota* compared with healthy controls (**Figure 2D**). At the genus level, LEfSe analysis with a linear discriminant analysis (LDA) threshold>2.5 revealed that the abundances of *Escherichia-Shigella* and the *Ruminococcus gnavus* group were significantly increased in the OA group, whereas the abundances of *Bacteroides*, *Eubacterium hallii* group, *Subdoligranulum*, and *Faecalibacterium* were significantly decreased (**Figure 2E**). The LEfSe cladogram showed differential taxa between the two groups from phylum to genus level, with the differential genera mainly belonging to *Firmicutes*, *Bacteroidetes*, and *Proteobacteria*. In summary, both the diversity and compositional structure of the gut microbiota are altered in OA patients.

### 3.4 Analysis of gut fungal microbiota in OA patients

**Figure 3:**
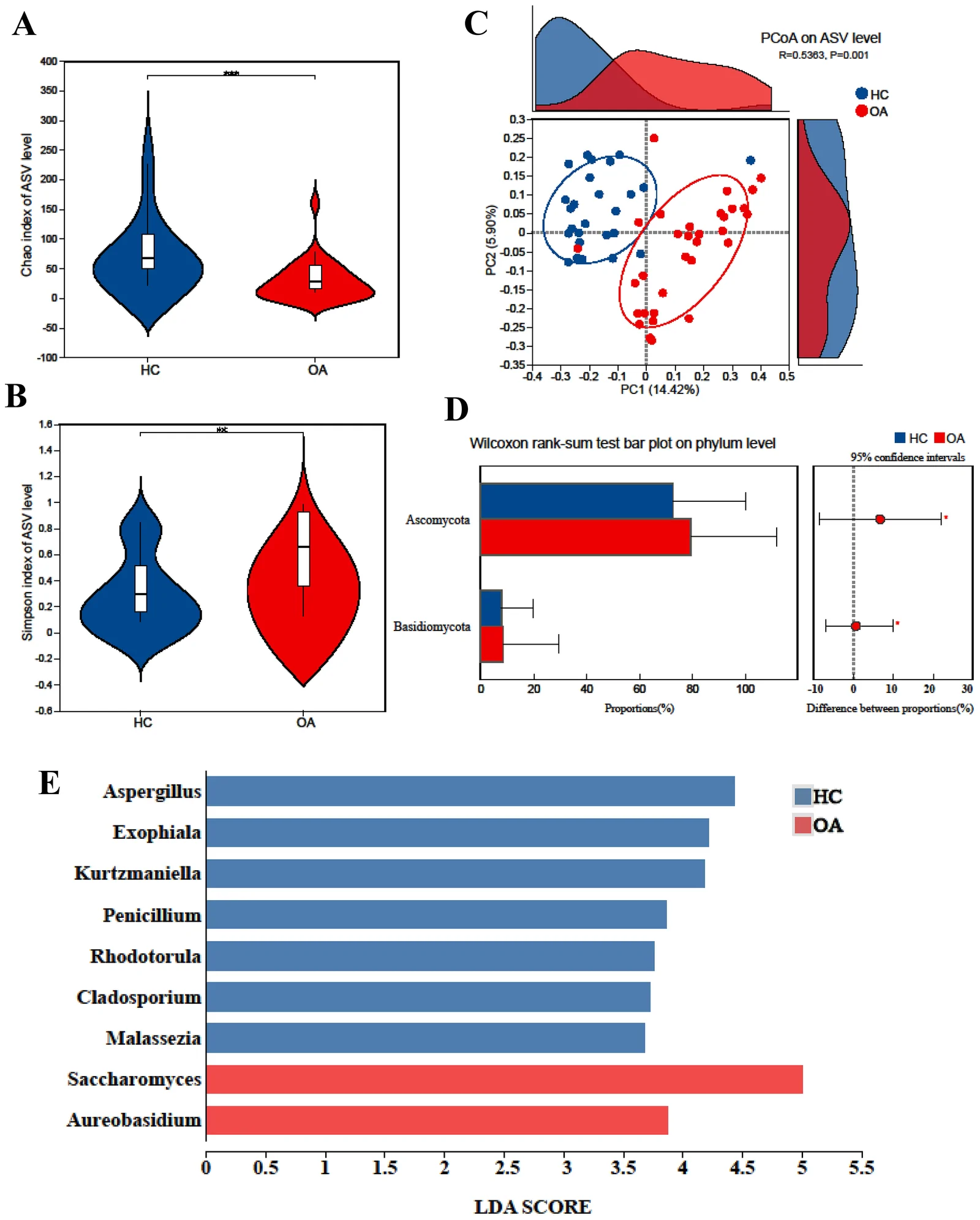
Comparison of gut mycobiota between the OA patients and healthy controls. **(A)** Chao index. **(B)** Simpson index. **(C)** PCoA analysis based on unweighted UniFrac distance at the ASV level. ANOSIM was used to analyze the differences in the gut fungal community between the OA group and the HC group. **(D)** Comparisons of the relative abundances of gut mycobiota at the phylum level between the OA and HC groups. **(E)** The LEfSe analysis of gut microbiota at the genus level between the OA and HC groups (LDA>3.5, *P* < 0.05). OA, osteoarthritis (n=32). HC, health control group (n=26). LEfSe, linear discriminant analysis effect size; LDA, linear discriminant analysis; Statistical differences were calculated using the Wilcoxon rank-sum test for two groups. *p < 0.05.

Changes in the gut fungal microbiota of OA patients were assessed using ITS2 amplicon sequencing. Alpha diversity was evaluated using the Chao and Simpson indices. Compared with healthy controls, the OA group exhibited a significantly lower Chao index (**Figure 3A**) and a significantly higher Simpson index (**Figure 3B**), indicating that gut fungal alpha diversity was significantly reduced in OA patients. PCoA based on unweighted UniFrac distances showed that samples from OA patients and healthy controls clustered in distinct regions of the coordinate space. ANOSIM revealed a significant difference between the two groups (R = 0.5363, *p* = 0.001), indicating a marked difference in gut fungal community composition between OA patients and healthy controls (**Figure 3C**). The Wilcoxon rank-sum test further analyzed the community structure. *Ascomycota* and *Basidiomycota* were the dominant phyla in both groups, and their relative abundances were significantly higher in the OA group than in the HC group (**Figure 3D**). At the genus level, LEfSe analysis revealed that the abundances of *Saccharomyces* and *Aureobasidium* were significantly increased in the OA group, while the abundances of seven genera, including *Aspergillus*, *Exophiala*, *Kurtzmaniella*, *Penicillium*, *Rhodotorula, Cladosporium*, and *Malassezia*, were significantly decreased (**Figure 3E**). The LEfSe cladogram showed differential taxa from phylum to genus level between the two groups, with these differential genera mainly belonging to *Basidiomycota* and *Ascomycota*. In summary, both the diversity and composition of the gut fungal microbiota are altered in OA patients.

### 3.5 Cross-kingdom network analysis of gut bacterial and fungal microbiota in OA patients

**Figure 4:**
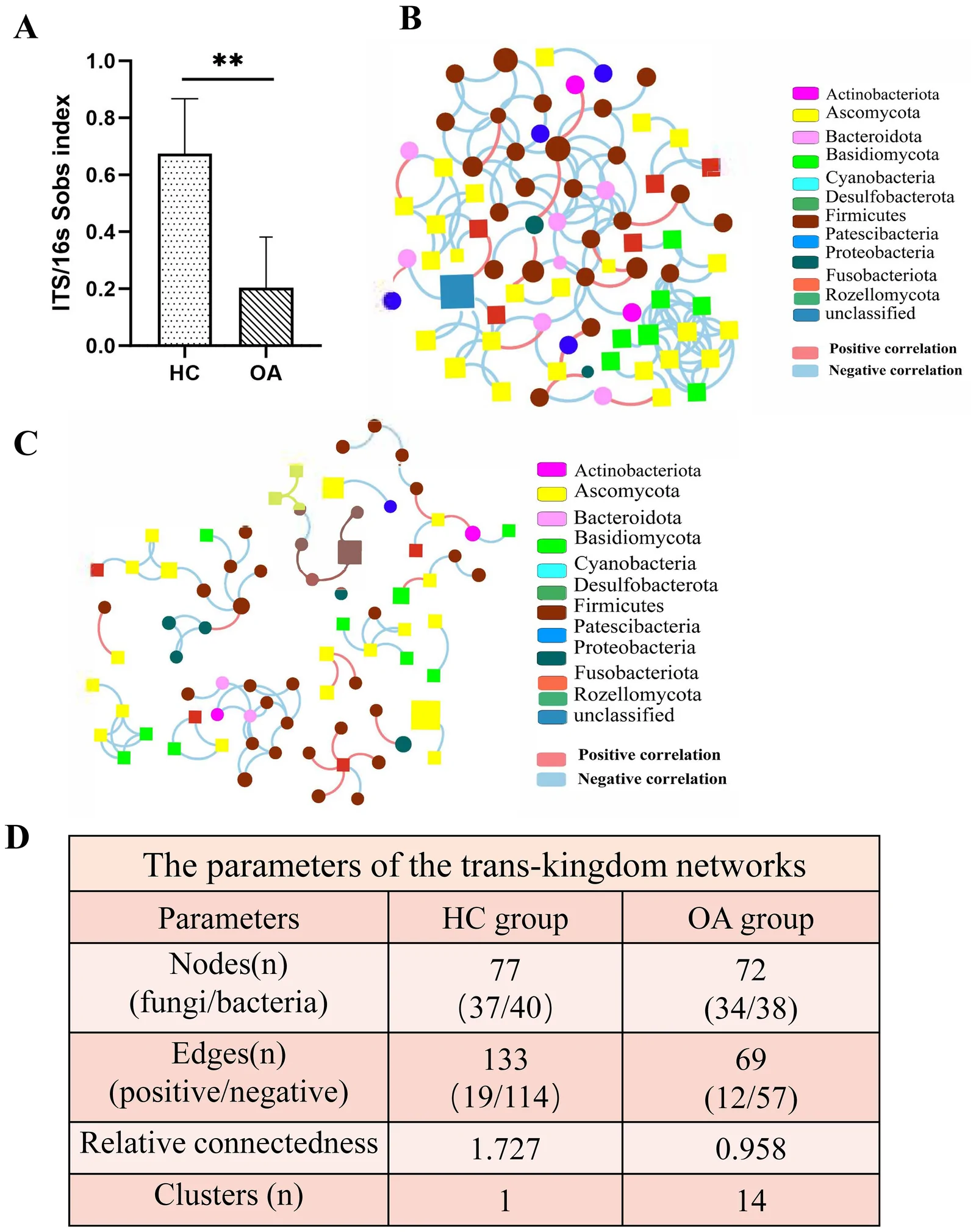
Fungal-bacterial equilibration analyses between the OA patients and healthy controls. **(A)** The ratio of ITS/16S at the Sobs index. The trans-kingdom abundance correlation networks of HC **(B)** and OA **(C)** groups at the genus level. In the network, bacterial and fungal taxa are depicted as circles and squares nodes, respectively, with distinct colors representing different bacterial genera. Red and blue edges denote positive and negative correlations, respectively. **(D)** The parameters of the trans-kingdom networks. Relative connectedness is defined as the ratio of the total number of edges to the total number of nodes in the network. OA, osteoarthritis (n=32). HC, health control group (n=26). Statistical differences were calculated using the Wilcoxon rank-sum test for two groups. **p < 0.01.

We assessed the overall balance of the gut microbiota by integrating both bacterial and fungal communities. Comparison of fungal and bacterial diversity using the ITS/16S Sobs index showed that this ratio was significantly lower in OA patients than in healthy controls (**Figure 4A**), indicating disruption of the balance between gut fungal and bacterial communities. Cross-kingdom network analysis revealed that in healthy controls, gut bacteria and fungi were tightly interconnected, forming a complex interaction network (**Figure 4B**). The correlation coefficient for bacterial–fungal interactions was 1.727 in the HC group (**Figure 4D**). In contrast, the cross-kingdom network in OA patients was sparser, with reduced interactions between bacteria and fungi and the formation of multiple dimers and trimers (**Figure 4C**). The correlation coefficient for bacterial–fungal interactions was 0.958 in the OA group (**Figure 4D**). These results indicate that OA reduces cross-kingdom interactions between gut bacteria and fungi, thereby disrupting the overall ecological balance of the gut microbial ecosystem.

### 3.6 Identification of characteristic gut microbiota in OA patients

**Figure 5:**
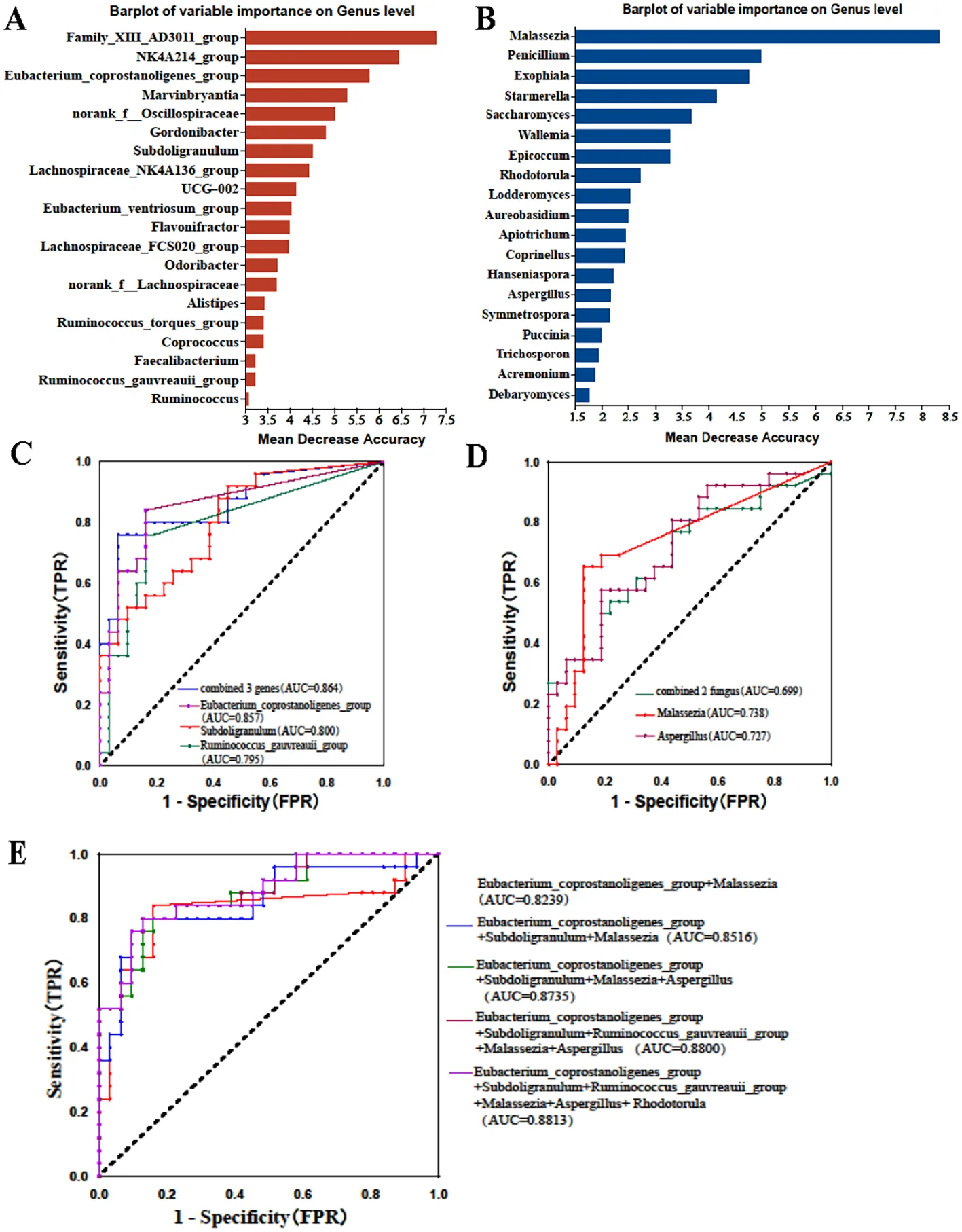
Analysis of the importance of gut microbiota in OA patients. **(A, B)** Variable importance rankings for bacterial **(A)** and fungal **(B)** constituents derived from Random Forest analysis. **(C, D)** Receiver operating characteristic (ROC) curves for the top-ranking bacterial **(C)** and fungal **(D)** taxa. **(E)** ROC curve for the combined bacterial–fungal panel. ROC, receiver operating characteristic; AUC, area under the curve (range: 0.5–1.0).

To identify characteristic gut microbiota that could serve as diagnostic biomarkers for OA, random forest analysis was performed on the two groups at the genus level. The analysis identified 20 characteristic bacterial genera, including *Family_XIII_AD3011_group*, *NK4A214_group*, *Eubacterium coprostanoligenes group*, and *Marvinbryantia* (**Figure 5A**). Additionally, 20 characteristic fungal genera were identified, including *Malassezia*, *Penicillium*, *Exophiala*, *Starmerella*, and *Saccharomyces*. Further selection based on differential abundance between the two groups identified six characteristic bacterial genera with significant differences. Among these, the *Ruminococcus gauvreauii group* and *Lachnospiraceae* were significantly enriched in the OA group, while *Ruminococcus*, the *Ruminococcus torques* group, *Subdoligranulum*, and the *Eubacterium coprostanoligenes* group were significantly decreased in the OA group. For fungi, seven characteristic genera with significant differences were identified, including *Malassezia*, *Penicillium*, *Exophiala*, *Rhodotorula*, *Aspergillus*, *Saccharomyces*, and *Aureobasidium* (**Figure 5B**).

ROC analysis was performed to validate the diagnostic potential of these characteristic microbial taxa (both bacterial and fungal). Three bacterial genera demonstrated potential to distinguish OA patients from healthy controls: *Eubacterium coprostanoligenes* group (AUC = 0.857, 95%CI:0.752-0.962), *Subdoligranulum* (AUC = 0.800, 95% CI: 0.686-0.914), and *Ruminococcus gauvreauii* group (AUC = 0.795, 95% CI: 0.670-0.919). The combination of these three bacterial genera showed stronger diagnostic performance (AUC = 0.864, 95% CI: 0.802-0.983; **Figure 5C**). Among fungal genera, ROC analysis showed that *Malassezia* (AUC = 0.738, 95% CI: 0.602-0.874) and *Aspergillus* (AUC = 0.727, 95% CI: 0.596-0.857) had moderate diagnostic value for OA; however, combining the two fungal genera reduced diagnostic performance (**Figure 5D**). The combination of three bacterial genera and two fungal genera exhibited superior diagnostic performance compared with using bacterial or fungal taxa alone (AUC = 0.880, 95% CI: 0.782-0.984; **Figure 5E**).

### 3.7 Correlation analysis between gut microbiota and inflammatory factors

**Figure 6:**
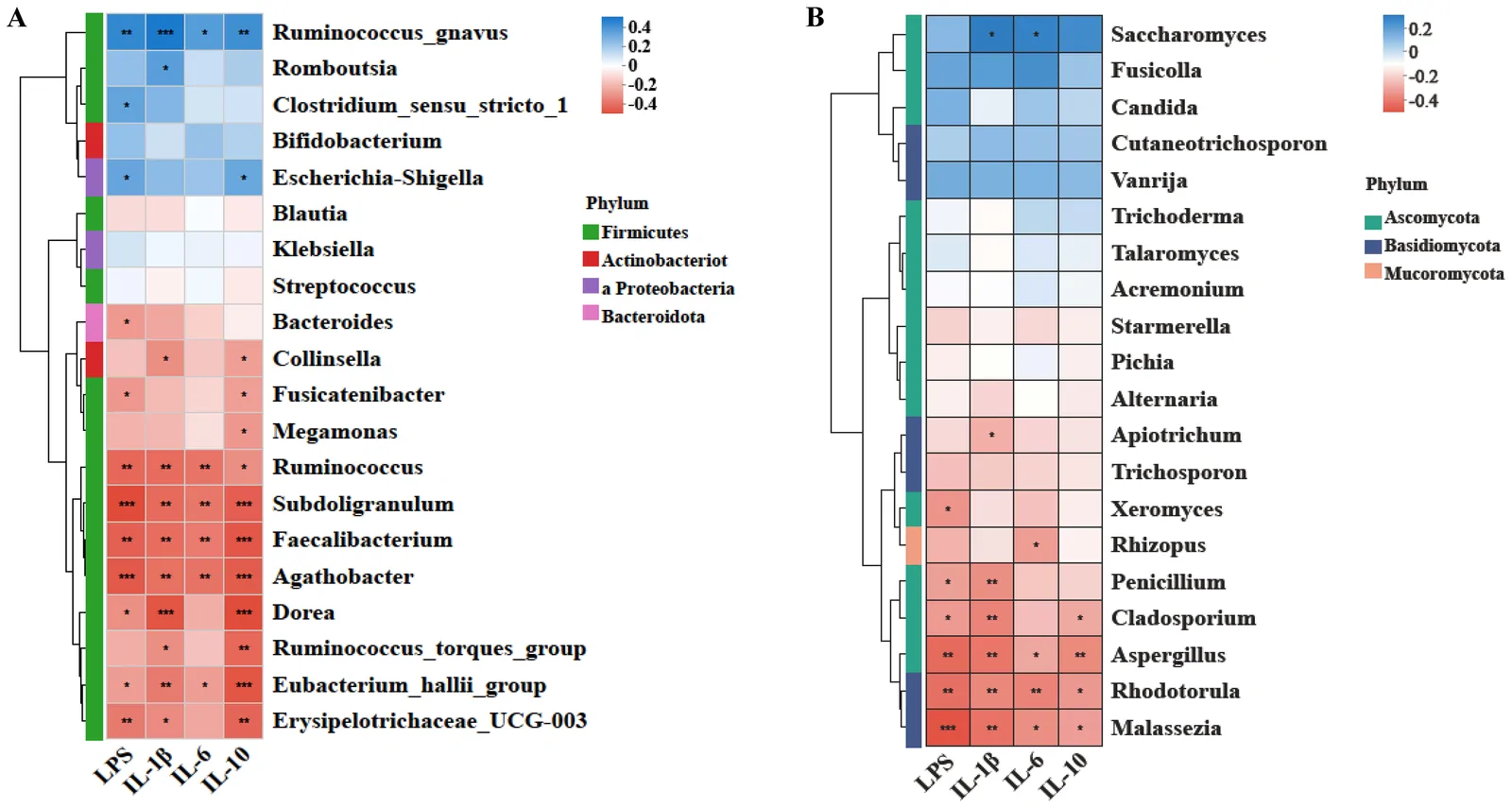
Correlation analysis between gut microbiota and inflammatory factors. **(A)** Heatmap depicting the correlations between the 20 most abundant gut microbial genera and serum inflammatory factors. **(B)** Heatmap illustrating pairwise correlations between the 20 most relatively abundant gut mycobiotal genera and circulating inflammatory factors (LPS, IL-1β, IL-6, and IL-10). Correlation coefficients were calculated using Spearman’s rank test, with color intensity representing the strength and direction of associations. *p < 0.05; **p < 0.01; ***p < 0.001.

To further explore the associations between inflammatory factors and gut microbiota, Spearman’s correlation analysis was performed using the 20 most abundant genera. The results showed that gut bacterial genera, including *Subdoligranulum*, *Agathobacter*, *Faecalibacterium*, *Ruminococcus*, and the *Eubacterium hallii* group, were negatively correlated with LPS, IL-1β, IL-6, and IL-10. In contrast, the *Ruminococcus gnavus* group was positively correlated with these inflammatory factors (**Figure 6A**). Among gut fungi, *Saccharomyces* was positively correlated with IL-1β and IL-6, whereas *Aspergillus*, *Rhodotorula*, and *Malassezia* were negatively correlated with LPS, IL-1β, IL-6, and IL-10 (**Figure 6B**).

**Figure 7:**
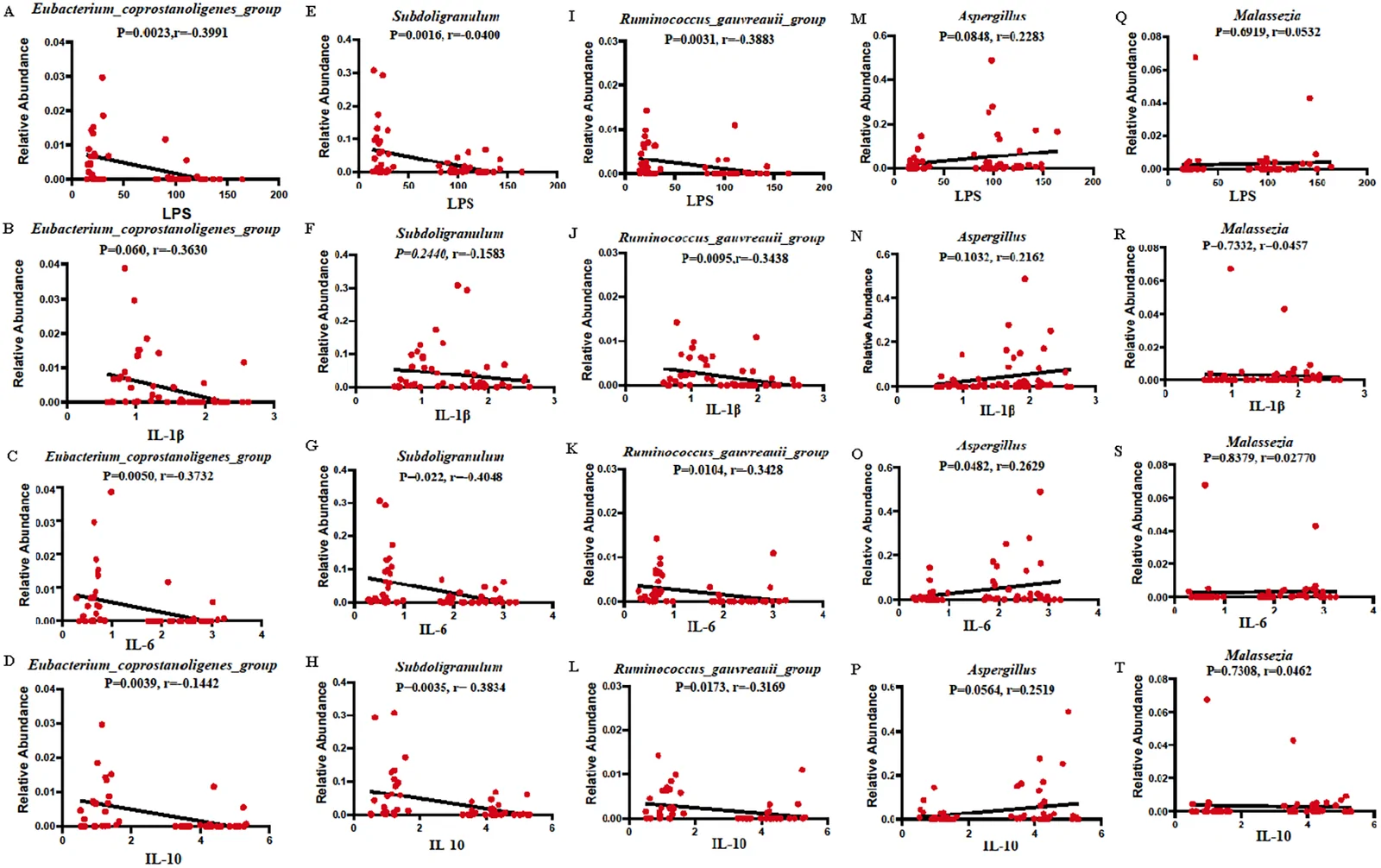
Linear Regression analysis of diagnostic biomarkers with serum inflammatory factors. Linear regression analysis between the relative abundance of five diagnostic biomarkers (*Eubacterium coprostanoligenes group*, *Subdoligranulum*, *Ruminococcus gauvreauii group*, *Aspergillus*, and *Malassezia*) and serum levels of four inflammatory factors (LPS, IL-1β, IL-6, and IL-10). **(A–D)** correspond to *E. coprostanoligenes group*; **(E–H)** to *Subdoligranulum*; **(I–L)** to *R*. *gauvreauii group*; **(M–P)** to *Aspergillus*; and **(Q–T)** to *Malassezia*, with each set of four panels representing the sequential regression against LPS, IL-1β, IL-6, and IL-10, respectively.

Subsequently, linear regression analysis was performed to evaluate the associations between the five diagnostic microbial genera and cytokine levels. The results showed that the *Eubacterium coprostanoligenes* group was negatively correlated with LPS, IL-1β, and IL-6 (R=-0.3991, -0.3630, and -0.3732, respectively; **Figures 7A–D**). *Subdoligranulum* was negatively correlated with IL-6 and IL-10 (R= -0.4048 and -0.3834, respectively; **Figures 7E–H**). The *Ruminococcus gauvreauii* group was negatively correlated with LPS, IL-1β, IL-6, and IL-10 (R= -0.3883, -0.3438, -0.3428, and -0.3169, respectively; **Figures 7I–L**). *Aspergillus* was positively correlated with LPS, IL-1β, IL-6, and IL-10 (r= 0.2283, 0.2162, 0.2629, and 0.2519, respectively; **Figures 7M–P**), while *Malassezia* showed weak correlations with these cytokines (**Figures 7Q–T**). Collectively, these data indicate that gut microbial dysbiosis in OA patients is associated with altered serum levels of LPS, IL-1β, IL-6, and IL-10.

## 4 Discussion

In this study, we compared the characteristics of the gut microbiota (including both bacterial and fungal communities) between OA patients and healthy controls. Our results showed reduced alpha diversity of the gut microbiota in OA patients, which is consistent with most previous studies (Dunn et al., 2020; Gilat et al., 2025). However, some studies have reported no changes in gut microbiota diversity between OA patients and healthy controls. Wei and colleagues, using 16S sequencing in the Xiangya Osteoarthritis Study, investigated the association between the gut microbiome and hand OA in 72 symptomatic and 1, 316 asymptomatic patients. They did not find any differences in alpha diversity but did observe increased alpha diversity of the genera *Bilophila* and *Desulfovibrio* and decreased alpha diversity of *Roseburia* in relation to hand OA (Wei et al., 2021). In the present study, we found that the abundance of *Proteobacteria* was significantly increased in OA patients, while the abundances of *Firmicutes* and *Bacteroidota* were significantly decreased. At the genus level, the abundances of *Escherichia*-*Shigella* and the *Ruminococcus gnavus* group were significantly increased in the OA group, whereas the abundances of *Bacteroides*, *Eubacterium hallii* group, *Subdoligranulum*, and *Faecalibacterium* were significantly decreased. Regarding the fungal microbiota, the abundances of *Saccharomyces* and *Aureobasidium* were significantly increased in the OA group, while the abundances of seven genera, including *Kurtzmaniella*, *Aspergillus*, and *Exophiala*, were significantly decreased. A survey of 1, 388 middle-aged and elderly Chinese patients showed decreased abundance of *Roseburia* and increased abundance of *Bilophila* and *Desulfovibrio* in OA patients (Wei et al., 2021). Another study reported significantly reduced abundance and diversity of gut microbiota in OA patients, such as decreased *Bifidobacterium longum* and *Faecalibacterium prausnitzii* and increased abundance of *Clostridium* (Chen et al., 2021). Wang et al. found increased abundances of *Fusobacteria* and *Bacteroidetes*, as well as higher abundances of *Alloprevotella*, *Robinsoniella*, and *Escherichia*-*Shigella*, in patients with rheumatoid arthritis (Wang et al., 2025). Some of these findings are consistent with our results, while others differ. It is well established that the gut microbiota is influenced by multiple factors, includingBMI, dietary habits, medication use, and comorbidities. To minimize the potential confounding effects of these variables on the present findings, we enrolled study participants who were closely matched for age, BMI, and geographical origin, thereby ensuring comparable dietary patterns (Individuals from the same geographic region were considered to have similar dietary patterns). In addition, patients with concurrent medication use or other concomitant diseases were excluded from the analysis. These measures were implemented to maximize the comparability of the study population. Despite these efforts, discrepancies between our results and those of previous studies may still be attributable to differences in sample size, geographic settings, and sequencing methodologies. Future investigations with expanded sample sizes will be required to further validate the observed alterations in the gut microbiota of patients with knee osteoarthritis.

Gut bacteria and fungi together constitute the gut microbiota. The abundances of and interactions between bacteria and fungi play important roles in maintaining intestinal barrier homeostasis. Disruption of the balance between gut bacteria and fungi may trigger inflammation. In this study, we found that the ITS/16S ratio was significantly lower in OA patients than in healthy controls, indicating disruption of the balance between gut fungi and bacteria in OA patients. Further analysis of gut fungal–bacterial interactions revealed a sparser cross-kingdom network in OA patients, with formation of multiple dimers and trimers, suggesting reduced interactions between gut bacteria and fungi. This result is consistent with another study. Research has also shown that alterations in the gut fungal microbiota and fungal–bacterial interaction networks are associated with knee synovitis (Jiang et al., 2023). Collectively, these findings suggest that gut fungi and bacteria should be evaluated as an integrated system when assessing their roles in disease pathogenesis.

The gut microbiome has been well utilized as a potential biomarker for the diagnosis of certain diseases. For some conditions, a single dominant taxon with relatively high abundance can serve as a potential biomarker (Duranti et al., 2016). Alternatively, combinations of multiple potential biomarkers can improve the efficiency of predictive models (Loomba et al., 2017; Han et al., 2024). In this study, we identified three bacterial genera, including *Eubacterium coprostanoligenes* group (AUC = 0.857), *Subdoligranulum* (AUC = 0.800), and *Ruminococcus gauvreauii* group (AUC = 0.795), and two fungal genera, including *Malassezia* (AUC = 0.738) and *Aspergillus* (AUC = 0.727), as characteristic microbial taxa for OA. ROC analysis indicated that these taxa could serve as potential diagnostic biomarkers for OA. When all five genera were combined as a diagnostic panel for OA, the AUC of the predictive model reached 88.13%. In another study, a combination of seven genera, including *Gemmiger*, *Klebsiella*, *Akkermansia*, *Bacteroides*, *Prevotella*, *Alistipes*, and *Parabacteroides*, as biomarkers for OA yielded an AUC of 83.36% (Wang et al., 2021). Although our predictive model demonstrates good diagnostic value, due to the relatively small sample size, there may be a risk of overfitting when constructing the ROC model. Therefore, in the future, a larger sample size is needed to verify the diagnostic efficiency and accuracy of our model.

In recent years, low-grade chronic inflammation has been recognized as playing a central role in the pathogenesis of OA. The source (or trigger) of this chronic inflammation is closely linked to gut microbiota. Existing studies have shown that disruption of the intestinal barrier allows gut microbes and their metabolites to leak into the systemic circulation, leading to endotoxin translocation and systemic inflammation, thereby promoting the onset and progression of OA (Lorenzo et al., 2019; Tsai et al., 2020). Gut microbiota play an important role in maintaining intestinal barrier integrity. Dysbiosis of the gut microbiota can lead to intestinal barrier damage and increased intestinal permeability. Our study showed gut microbiota dysbiosis and elevated serum LPS levels in OA patients, suggesting the occurrence of a “leaky gut, “ which may be a prerequisite for promoting OA development. Previous studies have shown that alterations in gut microbiota activate the innate immune system, leading to increased pro-inflammatory cytokines, thereby affecting OA pathogenesis at both systemic and local levels (Arend and Firestein, 2012; Kraus et al., 2016; Liu et al., 2019). One study found that the abundance of *Streptococcus* in the gut microbiota was positively correlated with knee pain. The researchers speculated that metabolites and vesicles produced by *Streptococcus* and its members may cross the gut–blood barrier and activate macrophages in the synovial intima, leading to joint inflammation and damage (Boer et al., 2019). Other studies have confirmed that obesity induces alterations in gut microbiota, with certain gut bacteria activating inflammation. Activated macrophages and other inflammatory cells migrate into adipose tissue, releasing TNF and other pro-inflammatory cytokines into the circulation, which exacerbates systemic inflammation and promotes OA development (McAllister et al., 2018; Dabke et al., 2019).

LPS is closely associated with the severity and inflammation of knee OA (Huang and Kraus, 2016). Studies indicate that LPS can activate macrophages and neutrophils in the innate immune system, inducing them to synthesize pro-inflammatory factors such as IL-1β, TNF, MMPs, and free radicals, leading to significant secondary inflammation in joint tissues (Lorenz et al., 2013). LPS not only initiates immune responses in joint tissue macrophages via TLR4 but also exacerbates existing chronic low-grade inflammation by upregulating pro-inflammatory cytokines (TNF, IL-1β, IL-6, etc.), thereby driving OA into a chronic disease state (Scanzello et al., 2008; Lorenz et al., 2013; Huang and Kraus, 2016). Furthermore, LPS can induce adipose tissue inflammation, promoting systemic alterations in cytokines, adipokines, and growth factors such as leptin, IL-10, and IL-1β, ultimately facilitating OA progression by influencing the local inflammatory environment within the knee joint (Bleau et al., 2015). Thus, LPS can activate the innate immune system through multiple pathways and mediate the expression of various cytokines, playing a pathogenic role in the persistent development of OA. In the present study, we also observed significantly elevated serum levels of LPS, IL-1β, IL-6, and IL-10 in OA patients, which is consistent with the findings described above.

In addition to the aforementioned mechanisms, gut microbiota dysbiosis may promote the pathogenesis and progression of OA through the disruption of host metabolic regulation and immune homeostasis. Age and obesity are well-established risk factors for OA. Previous studies have demonstrated that the elderly population exhibits a significant reduction in intestinal Bifidobacterium levels and a marked increase in the abundance of Aspergillus, which collectively elevate the susceptibility to intestinal barrier impairment. Consequently, in aging individuals, gut microbial imbalance is more prone to trigger immunosenescence and low-grade systemic inflammation, thereby facilitating the development of OA (Xi et al., 2025). Gut microbiota dysbiosis has also been implicated in the etiology of obesity. In obese patients, adipose tissue secretes adipokines such as leptin, which participate in metabolic regulation. Within OA chondrocytes, leptin binds to its receptor Ob-Rb and broadly activates multiple downstream signaling cascades, including the phosphatidylinositol 3-kinase/protein kinase B (PI3K/Akt), mitogen-activated protein kinase (MAPK), and related Janus kinase/signal transducer and activator of transcription (JAK/STAT) pathways, thereby driving inflammatory responses and increasing the risk of OA (Simopoulou et al., 2007; Xiong et al., 2019). Collectively, these findings indicate that gut microbiota dysbiosis contributes to the onset and progression of OA via multiple interrelated mechanisms.

Correlation analyses revealed that changes in LPS and cytokine levels were closely associated with alterations in gut microbiota. Therefore, modulating the gut microbiota to alter microbial composition and restore intestinal homeostasis may be a feasible approach to slowing OA progression. Several existing studies have attempted to alleviate OA development by regulating the gut microbiota. The use of prebiotics and probiotics has shown some efficacy in relieving OA symptoms (e.g., pain) and/or delaying pathological progression (joint degeneration) (Arora et al., 2021; Moyseos et al., 2024). Various strategies exist for regulating the gut microbiota. Precisely modulating the balance of gut microbiota in OA patients using specific methods remains a topic worthy of further investigation. A 6-month randomized controlled trial investigating oligofructose-enriched inulin probiotics in patients with knee osteoarthritis demonstrated that probiotic supplementation effectively alleviated clinical symptoms of the disease (Wang et al., 2026). In another clinical trial, a 3-month intervention with Bifidobacterium animalis subsp. lactis Probio-M8 significantly enhanced the efficacy of chondroitin sulfate in postmenopausal women with knee osteoarthritis (Wang et al., 2025). Additionally, a case report described a 20-year-old female patient with rheumatoid arthritis (RA) who, following fecal microbiota transplantation from an 8-year-old healthy donor, was able to gradually reduce her RA medication dosage, accompanied by marked improvements in rheumatoid factor titers, disease activity scores, and functional disability index (Zeng et al., 2020).

Several limitations of this study should be acknowledged. First, the relatively small sample size may have compromised the statistical power and robustness of the identified microbial markers. Second, the candidate microbial signatures observed herein have not yet been validated in an independent external cohort, which limits the generalizability of our findings. Thirdly, the internal validation procedures were not performed during the Random Forest model construction, which raise the possibility of overfitting and precludes an objective assessment of the model’s generalizabilit. Thus, future studies with larger, prospectively recruited cohorts and and rigorous internal validation protocols will be required to confirm these preliminary observations and to further elucidate the compositional changes in the gut microbiota associated with osteoarthritis.

In summary, this study identifies gut bacterial–fungal imbalance and reduced cross-kingdom interactions in OA patients. A five-genus panel comprising Eubacterium coprostanoligenes group, Subdoligranulum, Ruminococcus gauvreauii group, Malassezia, and Aspergillus exhibited good diagnostic accuracy for OA. Additionally, gut microbial dysbiosis was significantly associated with serum levels of LPS, IL-1β, IL-6, and IL-10 in these patients. The potential of modulating gut microbial balance as a therapeutic approach for OA warrants further investigation.

## Data Availability

The datasets presented in this study can be found in online repositories. The names of the repository/repositories and accession number(s) can be found below: https://www.ncbi.nlm.nih.gov/, PRJNA1479940.
